# Transcriptomics and metabolomics of blood, urine and ovarian follicular fluid of yak at induced estrus stage

**DOI:** 10.1186/s12864-024-10079-7

**Published:** 2024-02-21

**Authors:** Huangqing Zhao, Yongzhen Huang, Shi Shu, Guowen Wang, Changqi Fu, Rong Huang, Jun Zhang, Huawei Su, Yang He, Chuzhao Lei, Lei Du, Jiahao Zhao, Wei Peng

**Affiliations:** 1 Qinghai University, Xining, China; 2 Northwest A&F University, Yangling, Shaanxi, China; 3 China Agricultural University, Beijing, China

**Keywords:** Estrus, Yak, Metabolome, Transcriptome

## Abstract

To gain a deeper understanding of the metabolic differences within and outside the body, as well as changes in transcription levels following estrus in yaks, we conducted transcriptome and metabolome analyses on female yaks in both estrus and non-estrus states. The metabolome analysis identified 114, 13, and 91 distinct metabolites in urine, blood, and follicular fluid, respectively. The Kyoto Encyclopedia of Genes and Genomes (KEGG) analysis highlighted an enrichment of pathways related to amino acid and lipid metabolism across all three body fluids. Our transcriptome analysis revealed 122 differentially expressed genes within microRNA (miRNA) and 640 within long non-coding RNA (lncRNA). Functional enrichment analysis of lncRNA and miRNA indicated their involvement in cell signaling, disease resistance, and immunity pathways. We constructed a regulatory network composed of 10 lncRNAs, 4 miRNAs, and 30 mRNAs, based on the targeted regulation relationships of the differentially expressed genes. In conclusion, the accumulation of metabolites such as amino acids, steroids, and organic acids, along with the expression changes of key genes like miR-129 during yak estrus, provide initial insights into the estrus mechanism in yaks.

**Supplementary Information:**

The online version contains supplementary material available at 10.1186/s12864-024-10079-7.

## Introduction

Yak is a significant source of income for herdsmen in the Qinghai Plateau region, serving as the main economic breed [1]. They provide daily necessities such as meat, milk, and fur to local herdsmen [2]. Additionally, yaks were historically used as a means of transportation in ancient times when modern transportation was not developed. However, the development of the yak industry is hindered by breeding issues such as late sexual maturity, long calving interval, and unclear estrus period [3]. After calving, yaks typically exhibit estrus in the second year or later, rather than the year of calving, resulting in a prolonged postpartum period of low fertility [4]. The seasonal breeding characteristics of yaks also contribute to their low breeding efficiency, limiting the growth of the yak breeding industry [5]. Therefore, studying the yak estrus mechanism to improve breeding efficiency and promote yak production is of significant importance.

Estrus and ovulation in female animals are regulated by the neuroendocrine and reproductive hormones in the hypothalamic-pituitary–gonadal (HPG) axis system [6]. Within the HPG axis, gonadotropin-releasing hormone (GnRH) neurons are primarily located in the hypothalamus and act on the anterior pituitary to stimulate the release of luteinizing hormone (LH) and follicle-stimulating hormone (FSH) [7]. FSH and LH hormones then reach the ovaries via blood circulation, promoting ovarian development and inducing ovarian estrogen production [8]. Studies have shown that the regulation of hypothalamic-pituitary processes can be influenced by the use of exogenous GnRH, which in turn can affect physiological processes related to reproduction [9].

Omics, including genomics, transcriptomics, and other omics, are used to study the genome, transcriptome, and their objects, respectively [10]. In essence, the addition of "omics" to a molecular term implies a comprehensive or global assessment of a set of molecules [11]. Metabolomics, a branch of omics, involves the quantitative analysis of all metabolites in the body and aims to investigate the relationship between metabolites and physiological and pathological changes. As a relatively recent scientific research method, metabolomics has gained significant attention in yak research, particularly in the study of yak milk [12], yak blood [13], and yak meat [14]. Some studies have also explored the potential mechanisms of yak estrus through metabolomics. For example, Jia Zhou et al. used metabolomics and transcriptomics to study the follicles of yaks supplemented with NCG in their diets, revealing that an increased number of large ovarian follicles (diameter > 10 mm) were associated with 889 genes and 94 metabolites, which may contribute to the development of these follicles [15].

Understanding the molecular mechanisms underlying yak estrus development has been a prominent focus in yak genetic breeding research. Zhou et al. conducted metabolomic and transcriptomic analyses to identify the gene regulatory network and metabolic processes related to steroid hormone biosynthesis in ovarian tissue during follicular development, providing valuable insights into the regulation of metabolite changes and follicular development in yak estrus [15]. Hormone levels can objectively reflect the estrus state of yaks, making it essential to investigate hormone changes at the molecular level to study the estrus mechanism in yaks. Granulosa cells (GCs), as a crucial organ involved in animal estrus, secrete steroid hormones such as estrogen and progesterone, creating a crucial microenvironment for follicular growth [16]. In recent years, lncRNAs and miRNAs have emerged as hot topics in research. Constructing an interaction network among lncRNAs, miRNAs, and target mRNAs can shed light on their potential roles in GCs proliferation and differentiation.

In this study, we conducted a comprehensive analysis of the effects of sympathetic estrus treatment on yak estrus using transcriptomic and metabolomic data, aiming to uncover the underlying molecular mechanisms of the treatment on yak estrus.

## Materials and methods

### Ethics statement

The terms of all individual laboratory animals are approved by the Animal Policy and Welfare Committee of Northwest A&F University College (FAPWC-NWAFU, Protocol number WAFAC1008).

### Materials

For this study, we selected 10 non-pregnant female yaks of similar age and randomly divided them into two groups with 5 yaks per group. We employed a synchronous estrus treatment called Ovsynch, On day 0 of the experiment, the animals were injected with 100 µg of gonadotropin-releasing hormone (GnRH) and administered a progesterone vaginal insert. On day 7, they received an injection of 0.5 mg PG, and the vaginal insert was removed. On day 9, another 100 µg of GnRH was injected, and the yaks' estrus condition was observed [17].

Ten female yaks of the same age were raised in the yak breeding base in Gangcha County, Qinghai Province. After calving in May 2021, the postpartum yaks underwent a three-month isolation and weaning process starting in August 2021. Following a 10–12 day period of weaning and isolation for the calves, an estrus induction procedure was implemented. Then, we randomly selected five postpartum female yaks to undergo the Ovsynch protocol. We utilized an ovulation meter to determine the estrus status of yaks, where estrus values ranged from 200 to 300. Simultaneously, in conjunction with ultrasound diagnosis, we observed whether dominant follicles were present on the ovaries. If such follicles were identified, it indicated the occurrence of estrus. On the 10th day of the Ovsynch protocol, the estrus in yaks was confirmed, and the slaughtering process was carried out at Qinghai Xiahua Halal Meat Products Co., Ltd. We obtained ovaries from five yaks in estrus through the slaughtering process. Within 2 h of collection, the ovaries were placed in 35℃ physiological saline (0.9% w/v) and transported to the laboratory. After sterilization, follicular fluid was aspirated from the ovaries using a needle and syringe. Subsequently, granulosa cells were obtained by centrifugation at 1500 rpm [18]. Urine and blood samples were collected from live animals. We collected blood, urine, and follicular fluid from the estrus group (*n* = 5) and the control group (*n* = 5) under the same feeding conditions. Samples were rapidly cooled with liquid nitrogen and stored in a refrigerator at -80℃ for metabolomics sequencing and analysis. Additionally, we collected follicular granulosa cells from the control group (*n* = 3) and the estrus group (*n* = 3) and used the same storage method for RNA-seq.

### Untargeted metabolomics study

The metabolic profiling of urine, follicular fluidand bloodsamples was performed using the 2777C UPLC system (Waters, UK) coupled to the Xevo G2-XS QTOF (Waters, UK). ACQUITY UPLC HSS T3 column (100 mm*2.1 mm, 1.8 μm, Waters, UK) was used for separation.

ACQUITY UPLC HSS T3 column (100 mm*2.1 mm, 1.8 μm, Waters, UK) was used for separation. The column temperature was 40 °C and the flow rate was 0.5 ml/min. The mobile phase A consisted of water and 0.1% formic acid. B mobile phase consists of acetonitrile and 0.1% formic acid. The metabolites were eluted with the following gradient: 0–1 min, 99% mobile phase A; 1–3 min, 1–15% mobile phase B; 3–6 min, 15–50% mobile phase B; 6–9 min, 50–95% mobile phase B; 9–10 min, 95% mobile phase B; At 10.1–12 min, 99% mobile phase A was obtained. The loading volume of each sample was 5 μl.The small molecules eluted from the chromatographic column were respectively collected in positive and negative ion mode by high resolution tandem mass spectrometry Xevo G2-XS QTOF (Waters, UK).

### Metabolomics data analysis

We used Progenesis QI (version 2.2) to perform peak alignment, peak extraction, normalization, deconvolution, and compound identification. To calibrate the real sample signal, we utilized information from the QC sample and performed local polynomial regression fitting [19]. OmicStudio (https://www.omicstudio.cn/tool) was used for Principal component analysis (PCA), Partial Least Squares Discriminant (PLSDA), and volcano analysis. When PCA fell short in effectively distinguishing between inter-group samples, PLS-DA emerged as a compelling alternative. Going beyond its capacity for dimensionality reduction, PLS-DA also facilitated the efficient separation of sample categories. In addition to its role in reducing data dimensions, PLS-DA empowered the prediction of sample categories, making it well-suited for classification purposes. By constructing a classification prediction model, PLS-DA delved deeper into identifying the affiliations of additional samples, a task that exploratory PCA methods were unable to accomplish. We determined significantly regulated metabolites between groups by applying the following criteria: |log_2_(fold change)|≥ 1 and *P* valve < 0.05. The identified metabolites were then enriched using KEGG and Gene Ontology (GO) enrichment analysis on the MetaboAnalyst website (https://www.metaboanalyst.ca/).

### RNA extraction, sequencing of small RNA and LncRNA

We extracted total RNA using Trizol reagent (Ambion, United States) as per the manufacturer's instructions. Ligation PCR products were sequenced on the BGISEQ-500 platform (BGI Group, Shenzhen, China).

### MiRNA analysis and identification

To remove low-quality and adaptor sequences, we processed the data using SOAPnuke [20]. High-quality data obtained from the previous step were mapped to the Bos Taurus reference genome (ARS-UCD1.2_Btau5.0.1Y) using HISAT2 [21]. Due to the absence of miRNA sequences for yaks in the miRNA database, and considering the need for a consistent species in data analysis, the reference genome of yellow cattle was chosen. The miRNA sequences for yellow cattle were used for analysis. Data were compared with Rfam database (https://rfam.org/) to filter rRNA, scRNA, snoRNA, snRNA, tRNA and other types of non-coding small RNAs. We calculated the expression of miRNA using Bos Taurus in miRbase release 22.1 as a reference [22, 23]. Read counts were normalized by the total count of mappable reads of each sample, referred to as RPM, for unbiased comparisons among samples. Normalized expressions were further standardized by z-scores for cluster and PCA [24]. To study the expression of miRNA in the control group and the experimental group, we identified DE miRNAs using the edgeR package [25]. After differential expression analysis, we considered DE miRNAs with a range of |log_2_ (fold change)|≥ 1 and *P* valve < 0.05. We predicted the target genes of miRNA using miRanda [26], qTar, and RNAhybrid [27]. We performed the enrichment of GO and KEGG using DAVID Online with the target genes of miRNA [28].

### LncRNA analysis and identification

We processed and mapped lncRNA data using SOAPnuke and HISAT2, following the same approach we used for miRNA data. We used samtools mapping to convert the sam file to a bam file [29]. The transcript assembly and quantification were accomplished by merging all bam files into a complete GTF file using the default parameters and merge capabilities of StringTie [30]. StringTie then calculated the lncRNA expression FPKM (Fragments per kilo-base of exon per million fragments mapped) of the assembled GTF file using the "-e" and "-B" parameters. The identification of lncRNA was based on existing studies [31]. Gffcompare in StringTie was used to get the transcript assembly [32]. We used gffcompare in StringTie to preserve the intergenic transcripts with class codes "u," "x," "i," "j," and "o," according to StringTie specifications. We deleted transcripts with a single exon and less than 200 bp in length. Based on the location information of the exons in the remaining transcripts, we extracted and fused the genome sequences of corresponding exons into a complete transcript sequence. We evaluated the protein-coding potential of the transcripts using CNCI [33], CPC2 [34], and PLEK [35]. Transcripts that encode proteins were removed to preserve those that do not encode proteins. We translated the previously screened transcripts into possible protein domainjjs using Transeq [36] and compared the corresponding proteins in the Pfam database [37]. We removed those with significant hits (E-value < 1e-5) using HMMER [38].

### Prediction and annotation of lncRNA targets

LncRNAs function by acting on protein-coding genes through cis-acting elements and trans-acting factors. LncRNA trans target gene prediction is usually carried out when the sample size is large. We used Bedtools to predict the nearest target gene 100 kb upstream and downstream of the lncRNA [39]. We performed the enrichment of GO and KEGG using DAVID Online by the target genes of lncRNA.

### Constructing the targeting relationship between miRNA and lncRNA

By using Cytoscape_v3.7.2 software, differentially expressed mirnas were combined with lncRN, and the targeting relationship between relevant lncrnas and mirnas was constructed.

### ceRNA network construction

The Competing Endogenous RNA (ceRNA) regulatory network is a regulatory mechanism based on the mutual competition of RNA molecules. In this network, different types of RNA, such as mRNA, miRNA, and lncRNA, compete with each other for miRNA binding sites, thereby influencing each other's expression levels. This competitive relationship forms a complex regulatory network, believed to play a crucial role in the regulation of gene expression. We used Cytoscape_v3.7.2 software to construct a related cceRNA regulatory network by combining differentially expressed miRNAs and lncRNAs with mRNA predicted by the previous software.

### Joint analysis of metabolites and genes.

Building and Analyzing Fully Connected Metabolite and mRNA Networks Using MetScape v3.1.3, a Plugin for Cytoscape Software [40, 41]. MetScape aids in constructing networks of metabolites and genes, tracing their interconnections, and visualizing the composite network.

## Results

### Analysis of differences in metabolic profiles of blood, urine, follicular fluid

**Fig. 1:**
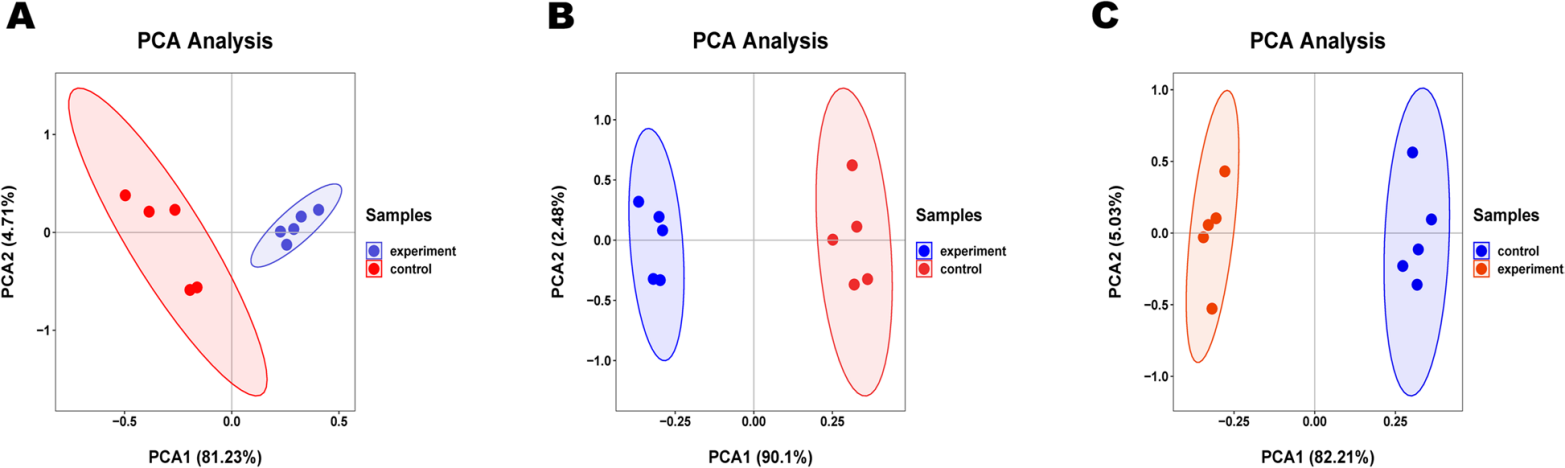
PCA of metabolites in urine, blood and follicular fluid in estrus group and control group. **A** Urine **B** Blood **C** Follicular fluid

**Fig. 2:**
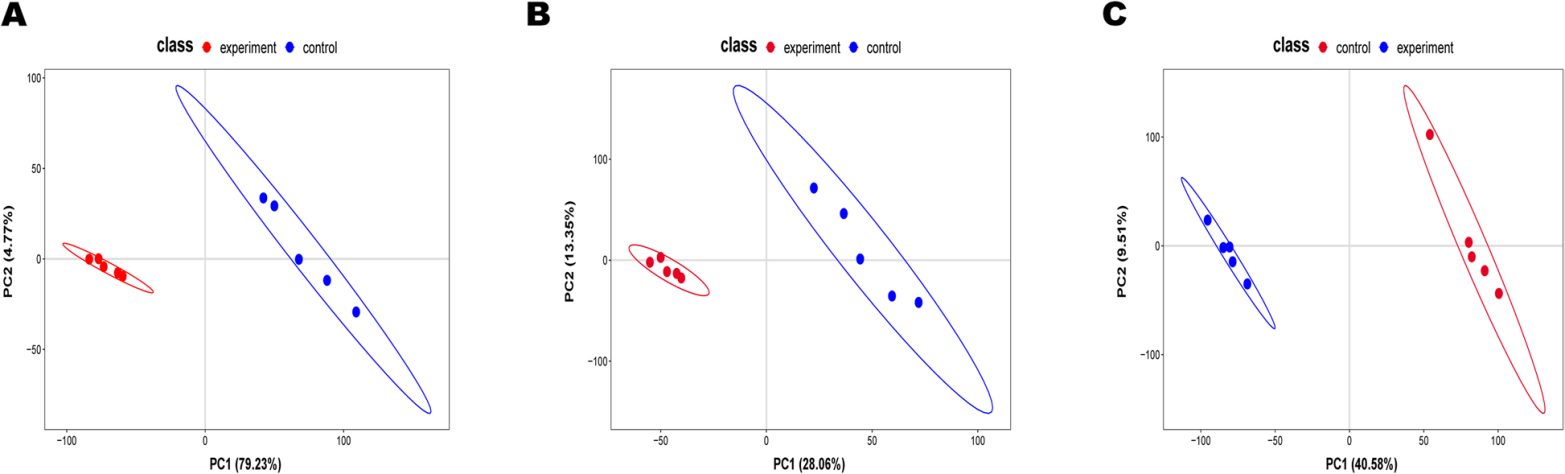
PLSDA of metabolites in urine, blood and follicular fluid in estrus group and control group. **A** Urine **B** Blood **C** Follicular fluid

**Fig. 3:**
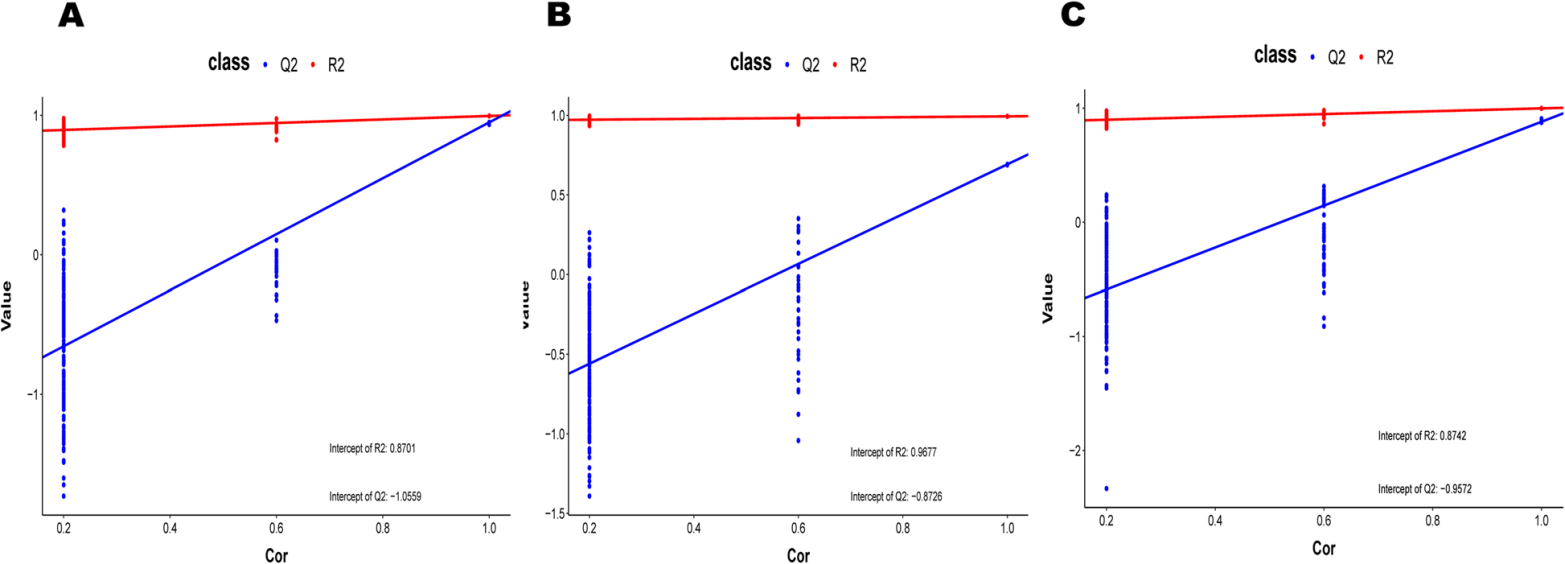
PLSDA replacement test of metabolites in urine, blood and follicular fluid in estrus group and control group. **A** Urine **B** Blood **C** Follicular fluid

The data of serum, follicular fluid, and urine samples obtained through LC–MS were integrated and analyzed using principal component analysis A (Fig. 1). In principal component analysis, the scoring maps of serum, urine, and follicular fluid samples showed significant changes in metabolites. On the basis of PCA analysis, the control group and blank control group of the three samples were subjected to multivariate data statistical analysis, and the PLS-DA model was used to further analyze and predict the inter group differences between different products (Fig. 2). The blank control group and control group were significantly separated, and the quality of PLS-DA was evaluated by two parameters R2 and Q2 (Fig. 3). Their explanatory power was good (R2), but their predictive power was poor (Q2), so the Variable Importance in Projection (VIP) cannot be used for screening differential metabolites.

### Screening of differential metabolites

**Fig. 4:**
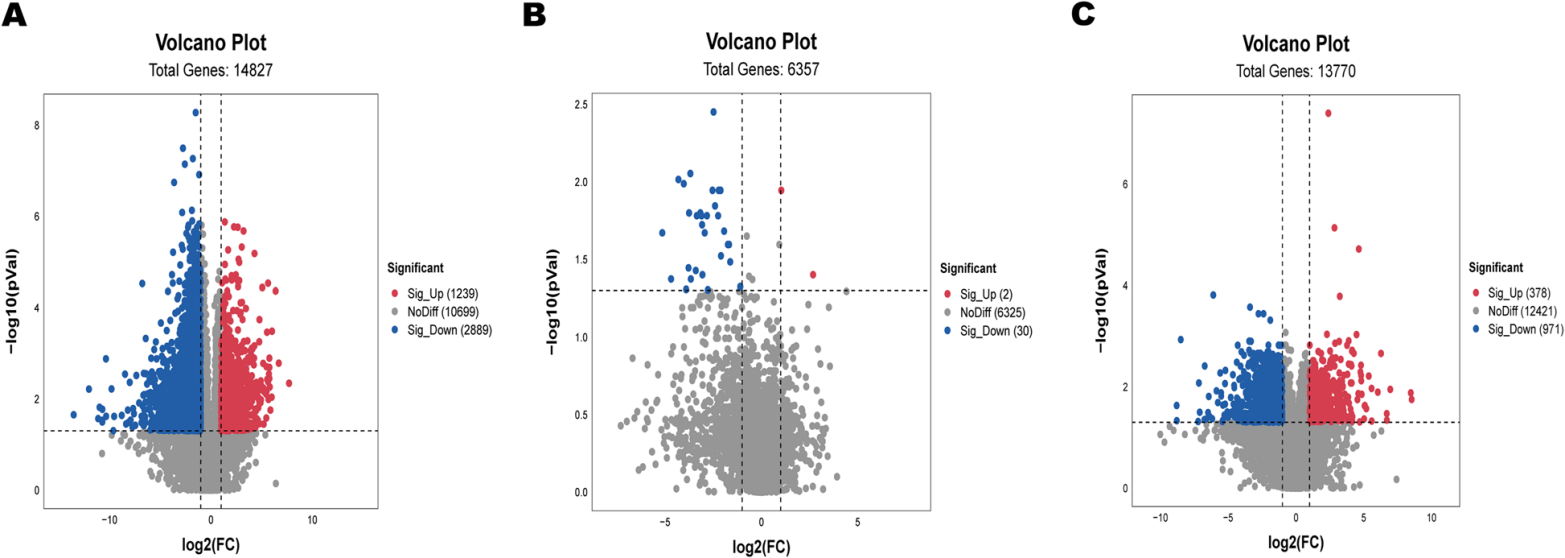
Volcanic plot in urine, blood and follicular fluid of estrus group and control group. **A** Urine **B** Blood **C** Follicular fluid

Differential metabolites were screened based on the following conditions: |log_2_ (fold change)|≥ 2 and *p*-value < 0.05. A volcano plot (Fig. 4) revealed 1239, 2, and 378 up-regulated metabolites in urine, blood, and follicular fluid, respectively, along with 2899, 30, and 971 down-regulated metabolites. However, these differential metabolites include isomers, which require enrichment into pathways for screening using the metaboanalyst website. Further screening is done based on the *P*-value (*P* < *0.05*) obtained from one-way analysis of variance during pathway enrichment. The metabolites annotated into significantly enriched pathways are considered as the differential metabolites of interest. A total of 114, 13, and 91 different metabolites were screened in urine, blood, and follicular fluid, respectively, with most of them being amino acids, steroids, and organic acids (Table S1, Table S2, Table S3).

### Pathway enrichment analysis of differential metabolites

**Fig. 5:**
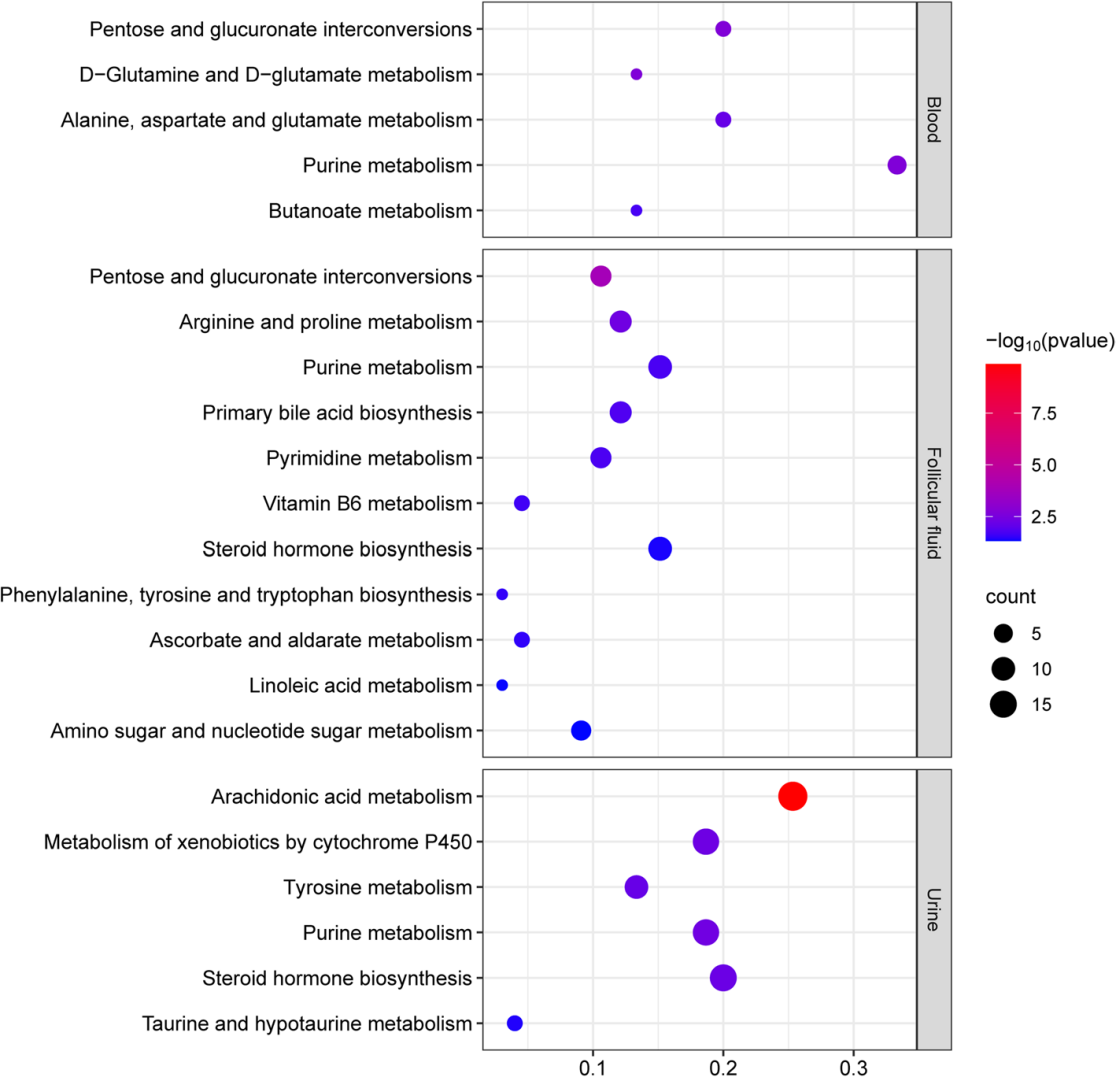
KEGG enrichment in urine, blood and follicular fluid of estrus group and control group

KEGG is a powerful tool for analyzing metabolism and conducting metabolic network research in vivo. Pathway enrichment analysis can help identify key biochemical metabolic pathways and signal transduction pathways associated with differential metabolites. In this study, KEGG pathway enrichment analysis was performed on differentially expressed metabolites in urine, blood, and follicular fluid from both the control group and the experimental group [42]. The results were visualized as bubble plots in Fig. 5. In the plots, the horizontal axis represents the number of differential metabolites in the corresponding metabolic pathway relative to the total number of identified metabolites in the pathway. A higher value indicates a greater enrichment of differential metabolites in the pathway. The color of the bubbles represents the *P*-value of the hypergeometric test, with smaller values (i.e., larger -log10(*P*-value)) indicating higher reliability and statistical significance. The size of the bubbles represents the number of differentiated metabolites in the corresponding pathway, with larger bubbles indicating a higher count of differentiated metabolites. In urine, the differential metabolites were mainly enriched in Arachidonic acid metabolism, Metabolism of xenobiotics by cytochrome P450, Tyrosine metabolism and other pathways. In follicular fluid, the differential metabolites were mainly enriched in Pentose and glucuronate interconversions, Arginine and proline metabolism, Purine metabolism and other pathways. In blood, the differential metabolites were mainly enriched in Pentose and glucuronate interconversions, D − Glutamine and D − glutamate metabolism, Alanine, aspartate and glutamate metabolism, and other pathways (Table S4).

### Overview of RNA sequencing data

We isolated follicular granulosa cells from yaks during estrus (*n* = 3, designated as group A) and from control yaks (*n* = 3, designated as group B) for RNA-seq analysis. The raw read counts for miRNA sequencing ranged from 28,915,662 to 43,636,363 reads, while the clean read counts ranged from 23,870,839 to 26,827,341 reads. The mapping read ratio varied from 70.09% to 85.08% across the six samples, and the Uniquely mapping ratio varied from 44.22% to 77.22%. For all samples, the Q20 score for clean tags was > 97.9% (RNA length is a crucial parameter for sRNA-seq, and we found that the majority of sRNAs were distributed between 20-23nt, with an average length range of 19.85–22.09 nt (Table S5). Regarding lncRNA sequencing, the clean read counts for the six samples ranged from 30,644,868 to 48,337,836 reads, and the GC content ranged from 51.52% to 55.69%. The Q20 score for clean tags was > 96.35% for all samples, the mapping read ratio varied from 91.42% to 92.72% across the six samples (Table S6).

### Identification of differentially expressed miRNAs

**Fig. 6:**
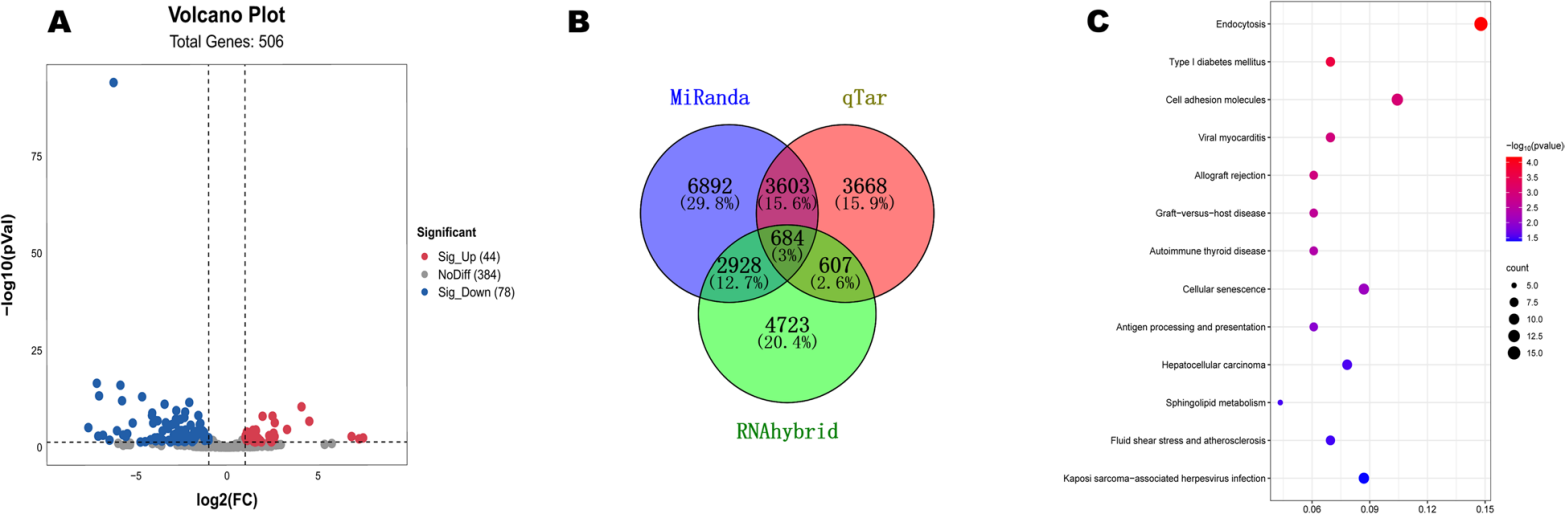
**A** Volcanic Plot of miRNA **B** Venn diagrams predicting miRNA target genes using three softwares. **C **KEGG analysis of target genes for differentially expressed miRNA

After conducting a thorough comparison of the Mirbase database, we obtained counts and performed differential expression analysis using the R-DESeq2 v1.14.1 version, employing a threshold of |log_2_(Fold Change)|> 1 and a significance level of *P* valve < 0.05. By visually representing the results with a volcanic plot (Fig. 6A), we identified 44 miRNAs that were up-regulated and 78 miRNAs that were down-regulated.

### Analysis of target genes for differentially expressed miRNAs

**Fig. 7:**
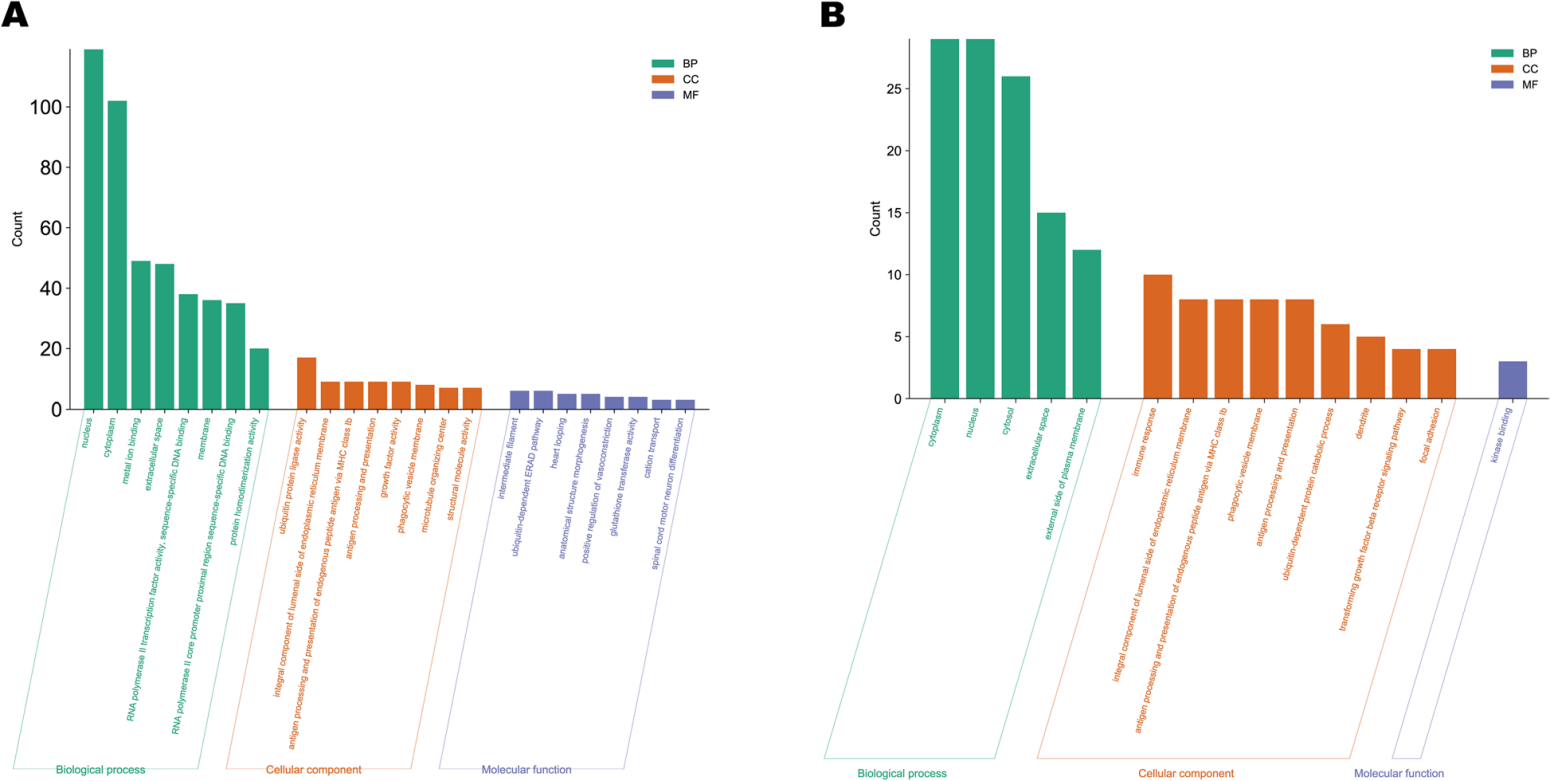
**A** GO analysis of target genes for differentially expressed miRNA. **B** GO analysis of target genes for differentially expressed lncRNA

The target genes of miRNAs were predicted using three different prediction software (miRanda, qTar, and RNAhybrid; Fig. 6B). Subsequently, GO and KEGG enrichment analysis were performed using the DAVID online website. The enrichment results of the KEGG pathway indicate that differentially expressed miRNA target genes are mainly enriched in Endocytosis, Type I diabetes mellitus, Cell adhesion molecules, Viral myocarditis and other pathways (Fig. 6C, Table S7). The GO functional annotation results show that in the biological process category, the main ones are cytoplasm, nucleus, cytosol and so on. In the cellular component, the main ones are immune response, integral component of lumenal side of endoplasmic reticulum membrane, antigen processing and presentation of endogenous peptide antigen via MHC class Ib and so on. In the molecular function, there is only a kinase binding (Fig. 7A, Table S8).

### Identification of differentially expressed novel lncRNAs

**Fig. 8:**
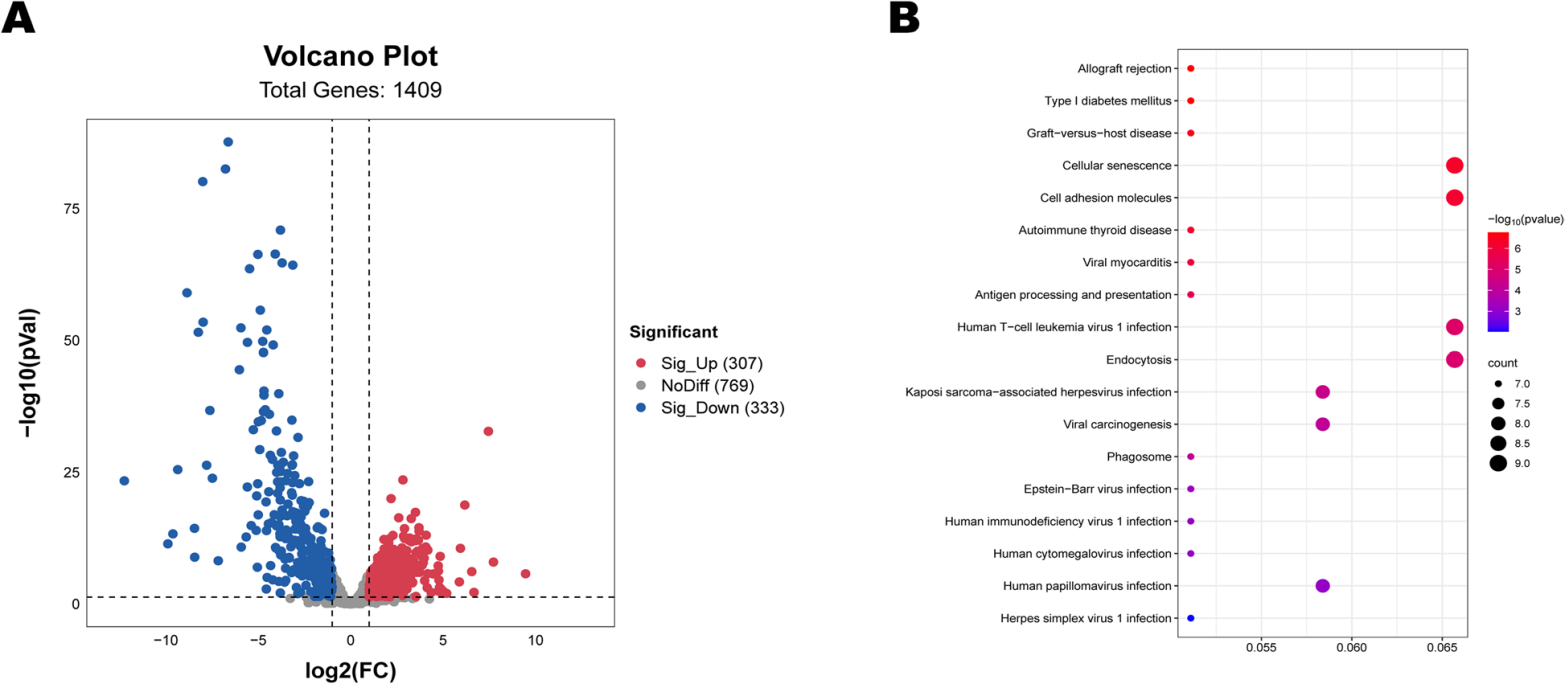
**A** Volcanic Plot of lncRNA **B** KEGG analysis of target genes for differentially expressed lncRNA

Following the quantification of counts obtained from the featurecounts software [43], we conducted differential expression analysis using DESeq R package version 1, with a threshold of |log_2_ (Fold Change)|> 1 and a significance level of *P* valve < 0.05. The outcomes were visually represented using a volcano plot (Fig. 8A), which enabled us to identify 307 up-regulated and 333 down-regulated lncRNAs.

### Analysis of target genes for differentially expressed lncRNAs

The target gene of lncRNA was predicted using the prediction software Bedtools. Subsequently, GO and KEGG enrichment analysis was performed using the same method as miRNA target gene analysis. The enrichment results of the KEGG pathway indicate that differentially expressed lncRNA target genes are mainly enriched in Allograft rejection, Type I diabetes mellitus, Graft − versus − host disease, Cellular senescence, Cell adhesion molecules and so on (Fig. 8B, Table S9). The GO functional annotation results show that in the biological process category, the main ones are cytoplasm, nucleus, cytosol. In the cellular component, the main ones are ositive regulation of transcription from RNA polymerase II promoter, nucleolus, ubiquitin-dependent protein catabolic process. In the molecular function, the main ones are BMP signaling pathway, calmodulin binding, positive regulation of protein phosphorylation (Fig. 7B, Table S10).

### Relationship between miRNA and lncRNA

**Fig. 9:**
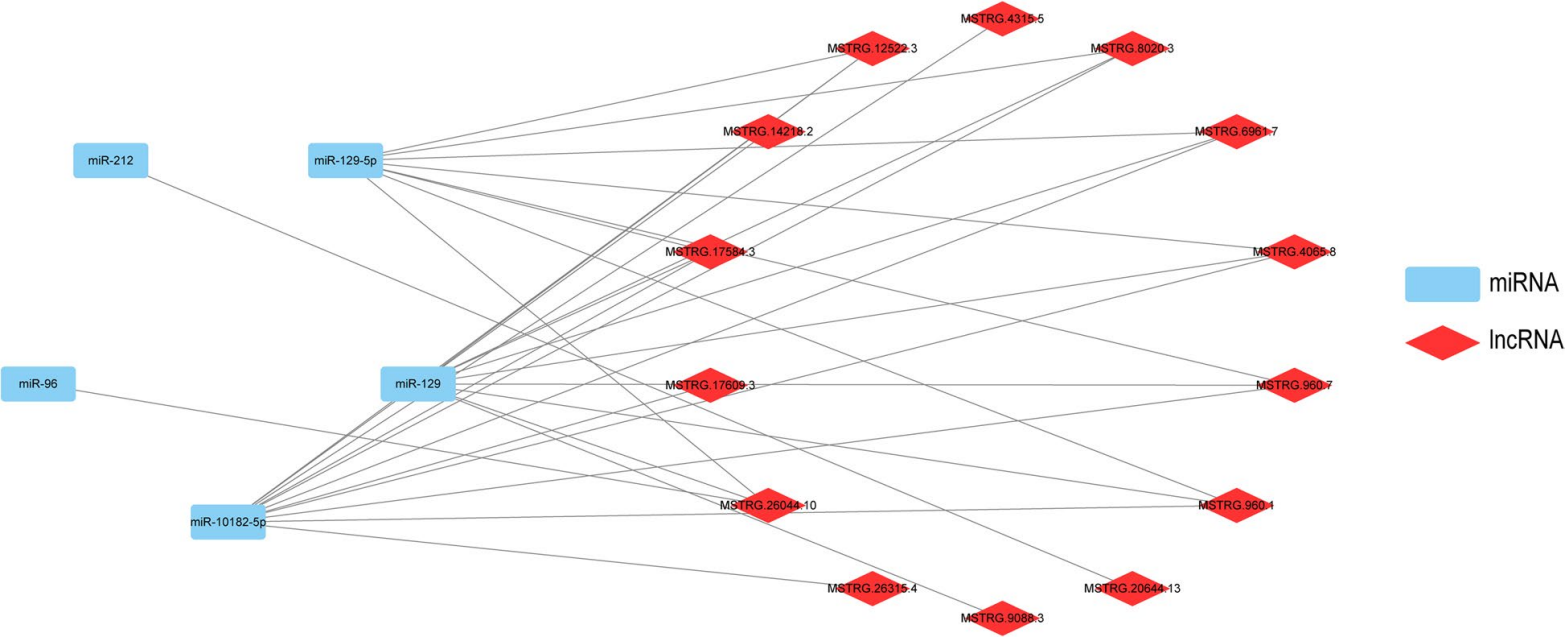
Relationship between miRNA and lncRNA

To better elucidate the targeting relationship between lncRNA and miRNA, we chose the top 5 miRNAs exhibiting the most significant differential expression. These were then paired with differentially expressed lncRNAs to construct a focused targeting relationship map, encompassing 5 miRNAs and 14 lncRNAs. This strategic approach aims to provide a clear and concise visualization of the intricate interactions within this subset of differentially expressed molecules (Fig. 9).

### Construction of ceRNA network

**Fig. 10:**
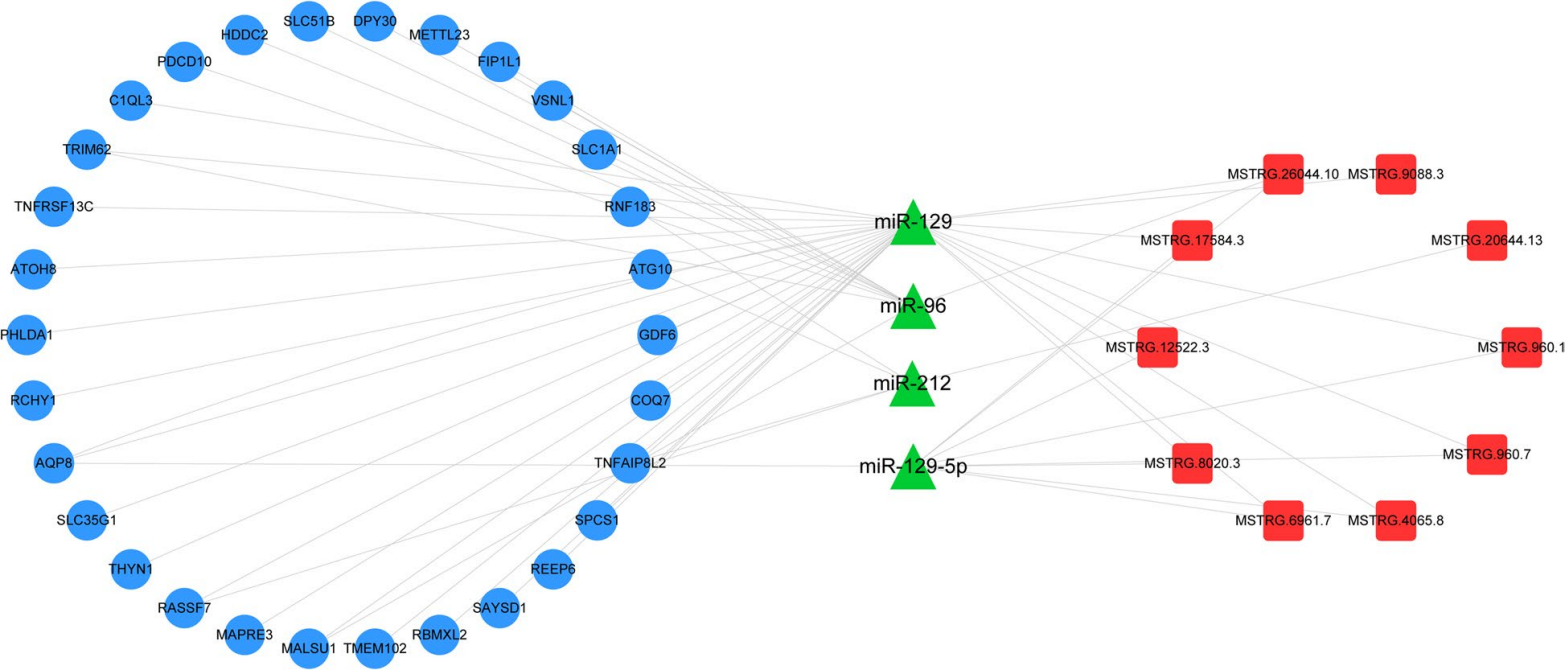
CeRNA Regulatory Network in Yak granulosa cells

lncRNAs can act as miRNA sponges, reducing their inhibitory effect on mRNA expression by sequestering miRNAs. A competing endogenous RNA (ceRNA) network involving lncRNAs and miRNAs was constructed based on differentially expressed lncRNA, miRNA, and predicted mRNA sequences. The ceRNA network comprised 44 nodes, including 10 lncRNAs, 4 miRNAs, and 30 mRNAs, and was visualized using Cytoscape software (Fig. 10). The ceRNA network revealed several mRNAs associated with estrus, such as GDF6 (Growth Differentiation Factor 6), TNFAIP8L2 (TNF Alpha Induced Protein 8 Like 2), COQ7 (Coenzyme Q7), SLC35G1 (Solute Carrier Family 35 Member G1), and AQP8 (Aquaporin 8). Additionally, several lncRNAs that interacted with miRNAs to exert ceRNA effects were identified, including MSTRG.960.1, MSTRG.960.7, MSTRG.4065.8, MSTRG.6961.7, MSTRG.8020.3, and others. These lncRNAs competitively inhibited miRNAs from binding to their corresponding mRNAs, thereby regulating mRNA expression.

### Network analysis of differential metabolites and genes.

**Fig. 11:**
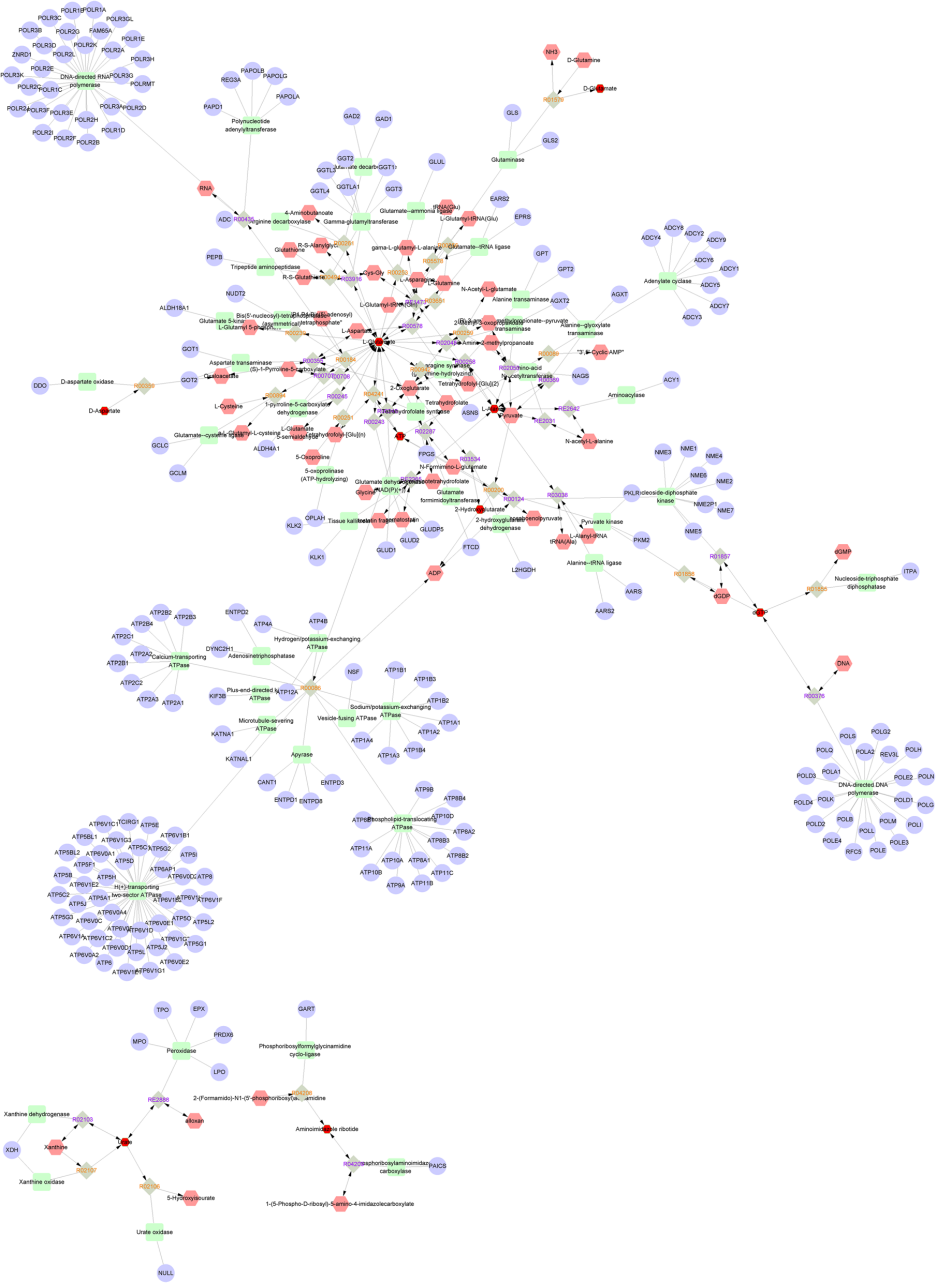
A network of metabolites and mRNAs in the blood

**Fig. 12:**
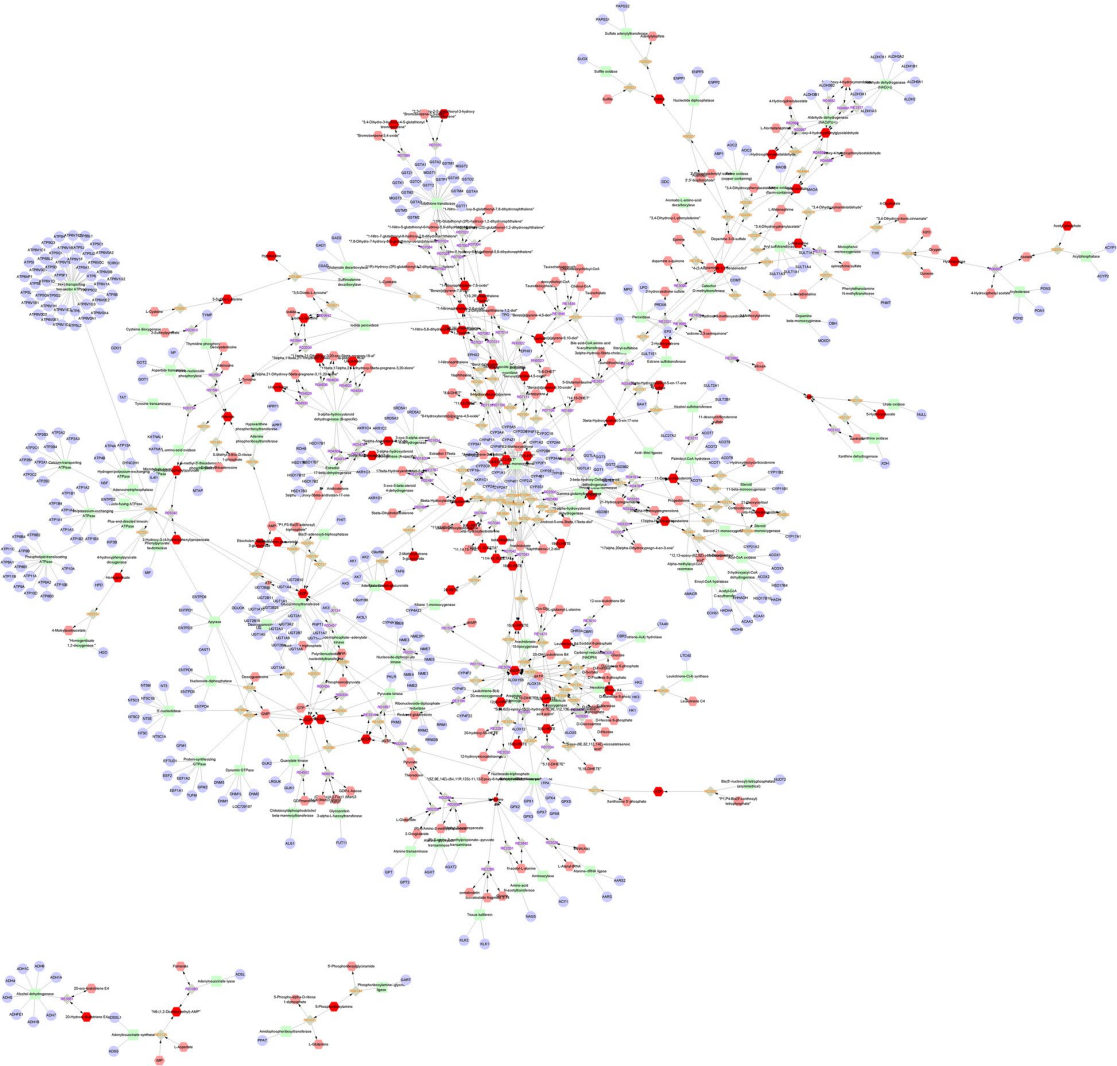
A network of metabolites and mRNAs in the urine

**Fig. 13:**
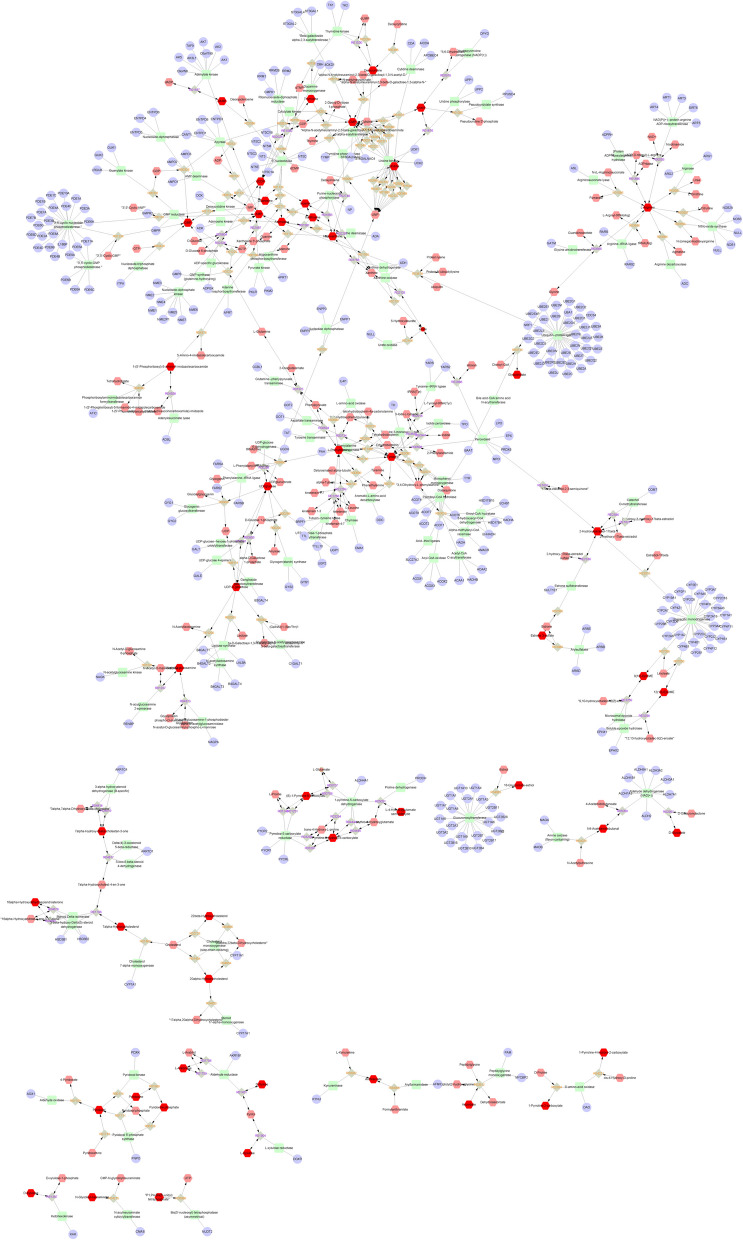
A network of metabolites and mRNAs in the follicular fluid

We used Metscape to construct a fully connected network of metabolites and mRNAs in urine, blood, and follicular fluid. Metabolites and mRNAs in the blood are mainly concentrated in the following pathways (Fig. 11): Glycine, serine, alanine and threonine metabolism, Glycolysis and Gluconeogenesis, Histidine metabolism, Purine metabolism, Urea cycle and metabolism of arginine, proline, glutamate, aspartate and asparagine, Vitamin B9 metabolism. Urine metabolites and mRNAs are mainly enriched to the following pathways (Fig. 12): Androgen and estrogen biosynthesis and metabolism, Arachidonic acid metabolism, Bile acid biosynthesis, C21-steroid hormone biosynthesis and metabolism, Glycine, serine alanine and threonine metabolism, Glycolysis and Gluconeogenesis and etc. on. Metabolites and mRNAs in follicular fluid are mainly enriched to the following pathway (Fig. 13): Aminosugars metabolism. Androgen and estrogen biosynthesis and metabolism, Bile acid biosynthesis, Biopterin metabolism, C21-steroid hormone biosynthesis and metabolism, Galactose metabolism, Purine metabolism, Pyrimidine metabolism, Tryptophan metabolism.

## Discussions

The yak is a unique breed of cattle that has been domesticated by highlanders living in the inhospitable high-altitude terrain of the central and inner Himalayas, serving as an indispensable breed for local herders in China. Despite its importance in the yak industry, the regulation of estrus in yaks is not well understood, with limited knowledge about the major endocrine and physiological mechanisms that may promote estrus in anestrus animals [44]. However, recent research on yak estrus is gaining momentum, with scholars conducting comprehensive analyses of the ceRNA network expression profiles of ovarian granulosa cells and cumulus cells in yaks, as well as transcriptome-wide analysis of N6-methyladenosine (m6A) to reveal the regulatory role of m6A in yak ovaries [45, 46]. In this study, we investigated the differentially expressed genes and differentially accumulated metabolites in the transcriptome and metabolome of estrous and unestrus yaks to explore the underlying mechanisms of estrus in yaks.

### Changes in primary metabolites related to estrus

Studies have demonstrated that estrus in mammals occurs due to the specific effects of ovarian steroid hormones on behavioral centers in the brain [47]. Metabolic levels of amino acids [48], fatty acids [49], proteins [50], and other substances undergo changes in animals during estrus. Urine, blood, and follicular fluid are important body fluid components and serve as crucial environments for detecting metabolites. Taking urine as an example, mammalian urine contains nonvolatile substances such as uric acid, urea, and protein, as well as volatile and semi-volatile substances such as short-chain fatty acids and species-specific signal communication molecules called pheromones [51]. Urine may play a significant role as a pathway for communication during mammalian estrus. Estrus-specific sex pheromones are believed to carry out chemical signaling in the presence of urinary protein ligands [52]. In our research, most metabolites are amino acids, steroids, and organic acids. In urine, the differential metabolites were mainly enriched in Arachidonic acid metabolism, Metabolism of xenobiotics by cytochrome P450, Tyrosine metabolism and other pathways. In follicular fluid, the differential metabolites were mainly enriched in Pentose and glucuronate interconversions, Arginine and proline metabolism, Purine metabolism and other pathways. In blood, the differential metabolites were mainly enriched in Pentose and glucuronate interconversions, D − Glutamine and D − glutamate metabolism, Alanine, aspartate and glutamate metabolism, and other pathways. Many studies have verified the effects of different metabolites in follicular fluid on follicular growth through oocyte culture in vitro.SINCLAIR et al. reported that glycine and alanine were the most abundant amino acids found in follicular fluid [53]. MATOBA et al. conducted a metabolomics analysis of in vitro cultured oocytes under normal and stop-growth conditions and found that glycine, L-alanine, and L-glutamic acid were positively correlated with follicular growth [54]. Fatty acids and steroids also play essential roles in animal estrus. Studies have shown that estradiol, progesterone, and arachidonic acid regulate estrus in pigs through PKA and MAPK signaling pathways [55]. Improving the nutritional status of female mammals through dietary fatty acid content has been shown to enhance ovarian function and follicle development in goats [56]. These findings suggest that primary metabolic pathways, such as lipid metabolism and amino acid metabolism, play a crucial role in yak estrus.

A series of changes occur in follicle development during yak estrus. The follicular fluid in the follicular cavity is the microenvironment of the oocyte. Follicular fluid provides nutrients for follicle development and contains hormones, growth factors, proteins, and phospholipids produced in part by granulosa cells (GC) [57]. Similarly, metabolites in follicular fluid, such as advanced glycosylation end products, have been found to improve the developmental potential of oocytes. Steroid metabolism in follicles appears to be closely related to ovarian function and oocyte quality [58, 59]. In addition, studies have shown that vitamin b6 supplementation can significantly increase luteinizing hormone (LH) production in sows during estrus [60, 61]. This is similar to the types of differential metabolites and enrichment pathways in our findings.

### Effect of miRNA on yak estrus

MiRNAs, which are a class of small non-coding RNAs, have been found to be closely associated with the growth and development of plants and animals in various studies [62]. Numerous studies have investigated the relationship between miRNAs and mammalian estrus. For instance, the interaction between miR-370-3p and target genes has been shown to play a crucial role in the reproductive regulation of small-tailed Han sheep, participating in the post-transcriptional processes of follicular and luteal stages in different genotypes of small-tailed Han sheep [63]. Yongfu La and their colleagues have also identified miRNAs that are associated with testicular development in male yaks [64], shedding light on the role of miRNAs in regulating testis development and improving reproductive performance in male yaks. Additionally, A E Zielak-Steciwko et al. have identified miRNAs that are related to bovine follicular development and found that miR-383 may serve as an important biological regulatory factor controlling the growth and proliferation of dominant follicles [65].

In our research, we investigated the miRNAs of follicular cells involved in yak estrus using the RNA-seq technique. Interestingly, we observed that the small RNA lengths were predominantly concentrated in the range of 21–23 nucleotides, which is similar to the typical size of miRNAs. Through a volcanic plot, we identified 44 up-regulated miRNAs and 78 down-regulated miRNAs, some of which are highly expressed during mammalian estrus, such as miR-129, miR-129-5P, and miR-183, suggesting their potential widespread involvement in yak estrus. The roles of these highly expressed miRNAs in estrus have been studied, and it has been found that a group of differentially expressed miRNAs identified by miR-182-5P may regulate follicle formation through apoptosis, proliferation, and differentiation of follicular cells, ovarian steroid production, and oxidative stress [66]. Furthermore, the miR-183 cluster has been found to be significantly differentially expressed in preovulation dominant follicles, potentially playing a role in the post-transcriptional regulation of bovine follicular development genes during the late follicular phase of the estrous cycle [67]. These highly expressed miRNAs are believed to play a crucial role in yak estrus and should be given more attention in future research. Most miRNAs bind to mRNAs and regulate gene expression, and by predicting the target genes of these miRNAs, we have identified some target genes that play important roles in estrus.

The enrichment of KEGG and GO is a common analysis method used to demonstrate the effects of miRNA target genes. GO enrichment analysis revealed that the target genes of most differentially expressed miRNAs were involved in seven molecular functions, eight cellular components, and 10 biological processes, including microtubule, ATPase activity, and protein phosphorylation, among others, suggesting strong regulatory effects of these miRNAs on cell metabolism, motility, intracellular and intercellular signaling, molecular function, and protein catalytic activity during ovarian development and hormone secretion during estrus. Additionally, the enrichment results of the KEGG pathway analysis indicated that differentially expressed miRNA target genes were mainly enriched in pathways such as Endocytosis, Type I diabetes mellitus, Allograft rejection, Viral myocarditis, and Cell adhesion molecules. After ovulation, progesterone triggers decidualization of endometrial stromal cells, resulting in cytoskeletal changes. This critical process regulates embryonic invasion and establishes the necessary cytokine and immunomodulator environment in the stromal tissue during invasion. Consequently, cell adhesion molecules play a pivotal role in ovarian maintenance [68].

### Effect of lncRNA on yak estrus

lncRNA, a type of RNA molecule that exceeds 200 nucleotides and does not encode proteins, has been recognized for its diverse roles in various biological processes, making it a prominent area of research in the field of RNA in recent years. Recent studies have shed light on how lncRNAs can regulate gene expression at different levels, including epigenetic, transcriptional, and post-transcriptional regulation, and participate in important regulatory processes such as genome imprinting [69]. Growing evidence supports the presence and role of lncRNAs in reproductive tissues. For instance, Su and colleagues utilized high-throughput sequencing to identify differentially expressed lncRNAs during early pregnancy in Meishan and Yorkshire pigs, revealing that XLOC-2222497 and its target gene AKR1C1 can interact with progesterone in pig endometrium to regulate pregnancy maintenance [70]. Chen et al. identified 40 differentially expressed lncRNAs in the hypothalamus of sheep with different FecB genotypes during follicular and luteal phases [71]. In our own research, we identified 44 up-regulated and 78 down-regulated miRNAs, which were visualized using volcano plots. Studies have shown that lncRNA expression can regulate the expression of neighboring mRNAs [72]. Thus, we employed bedtools software to search for target genes located within 100 kb upstream and downstream of lncRNAs to predict the function of these lncRNAs. It is possible that lncRNAs act on adjacent or complementary target genes, playing a critical role in yak estrus. For example, MSTRG.73, a highly expressed lncRNA, targets the TIAM1 gene, which has been implicated in the invasive behavior of ovarian cancer, and down-regulation of TIAM1 is associated with increased aggressiveness of ovarian cancer cells [73]. Another target gene, MAP4K4, of MSTRG.2494, has been linked to the development of premature ovarian failure due to gene rearrangement [74]. Based on the function of these target genes, it is believed that these highly expressed lncRNAs may play a crucial role in yak estrus, and further investigation by researchers is anticipated in the future. Through the function of target genes, these highly expressed lncRNAs are believed to play a key role in yak estrus, which is expected to be further studied by scholars in the future.

The GO functional annotation results show that in the biological process category, the main ones are cytoplasm, nucleus, cytosol. In the cellular component, the main ones are ositive regulation of transcription from RNA polymerase II promoter, nucleolus, ubiquitin-dependent protein catabolic process. In the molecular function, the main ones are BMP signaling pathway, calmodulin binding, positive regulation of protein phosphorylation. Animals in estrus secrete hormones, and enzymes are needed for various physiological processes in estrus organisms, resulting in enhanced transcription, which is consistent with the biological processes of go enrichment analysis. The enrichment results of the KEGG pathway indicate that differentially expressed lncRNA target genes are mainly enriched in Allograft rejection, Type I diabetes mellitus, Graft − versus − host disease, Cellular senescence, Cell adhesion molecules and so on. The role of estrogen in the body relies on the transmission of signals between neurons and cells. Research indicates that estrogen can stimulate endocytosis of the postsynaptic membrane of neurons, resulting in increased density of small particles in the membrane and subsequently reducing the number of small particles. This process seems to be the physiological mechanism by which neurons in females change during the estrus cycle [75].

Recent literature reports suggest that the mutual regulatory network involving miRNA, lncRNA, and mRNA may also participate in the estrus process of yaks [45]. In the ceRNA network of yak follicular cells during estrus, a total of 10 lncRNAs, 4 miRNAs, and 30 mRNAs were identified. miR-129-5p is a representative miRNA [66], and lncRNA of MTSRG.26652 serves as a representative of the lncRNA group [76]. MRNA of GDF6 is also involved in animal estrus [77]. miR-129 is a key miRNA, and the nodes in the ceRNA network directly or indirectly participate in yak estrus. The role of the miR-129 family in animal ovarian estrus has been a research hotspot in recent years, and mir-129-5p, as a member of this family, has been shown to affect follicular development in sheep under the stimulation of FSH [66].

### Association analysis of transcriptomics and metabolome

Metabolomics and transcriptomics are potent omics technologies that yield comprehensive metabolite and transcript profiles [78]. The study of transcriptomics and metabolomics is in full swing in animal husbandryrespectively. In a recent investigation of Qinchuan cattle, a holistic examination involving blood transcriptomics and metabolomics was undertaken to unveil the molecular mechanisms regulating backfat thickness. The study revealed 1106 differentially expressed genes and 86 differentially expressed metabolites in cattle with varying backfat thickness. Noteworthy among these were the functional genes *SMPD3* and *CERS1,* along with the metabolite sphingosine 1-phosphate, identified as pivotal factors influencing phenotype variations. These results underscore the efficacy of integrating metabolomics and transcriptomics in livestock research, offering valuable insights for enhancing beef performance through informed breeding strategies [79]. In a recent study on pigs, researchers investigated the impact of miR-149-5p on intramuscular fat deposition using a comprehensive approach involving metabolomics and transcriptomics. The study revealed that miR-149-5p promoted the proliferation of porcine intramuscular preadipocytes while concurrently reducing their differentiation ability. Metabolomics analysis further demonstrated significant alterations in lipid, organic acid, and organic oxygen metabolites upon the overexpression of miR-149-5p in porcine intramuscular preadipocytes [80]. In a separate inquiry, an integrated analysis of liver metabolomics and transcriptomics was conducted to unveil oxidative stress in piglets affected by intrauterine growth restriction. The study highlighted a range of metabolic abnormalities in intrauterine growth-restricted piglets, including mitochondrial dysfunction, imbalances in fatty acid composition, disruptions in one-carbon unit supply sources, and abnormal galactose conversion. These irregularities are suggested to contribute to oxidative stress in the liver [81].

In our study, we employed MetScape software to construct and analyze metabolic and mRNA networks in urine, blood, and follicular fluid. In the blood (Fig. 11), the two most enriched pathways for metabolites and mRNA were the urea cycle and the metabolism of arginine, proline, glutamic acid, aspartic acid, and asparagine, along with purine metabolism. The role of purine metabolic pathways in reproduction remains unclear [82]. In the present investigation, purines and pyrimidines served as precursors for DNA and RNA biosynthesis [83], playing a crucial role as genetic material in normal cellular functions [84, 85]. During the estrous cycle of female animals, we hypothesized that cells might have needed to synthesize new DNA and RNA to support the development of ova and the division of reproductive cells, potentially achieved through the activation of purine metabolism. Purines could undergo a series of reactions to be broken down into uric acid, which was then excreted through urine. In our differential metabolite analysis, the levels of purines in the estrus group decreased compared to the control group, aligning with our speculative hypothesis. In the urine (Fig. 12), the gene most prominently associated in our study is the 17β-hydroxysteroid dehydrogenase family. The 17β-hydroxysteroid dehydrogenase family (17β-HSD) constitutes a group of enzymes that catalyze the conversion of 17-ketosteroid steroids into biologically more potent estrogens and androgens. For instance, they facilitate the conversion of estrone (E1) to the more active estradiol (E2) and the transformation of androstenedione into testosterone [86]. The 17β-hydroxysteroid dehydrogenase 12 (HSD17B12) has been demonstrated to play a role in the regulation of the prostaglandin synthesis pathway, ovarian function, and fertility. The HSD17B12 enzyme exhibits widespread expression in both human and murine ovaries. In mice, heterozygous deletion of the *HSD17B12* gene results in subfertility, indicating its significant role in ovarian function. Heterozygous female mice with a deficiency in HSD17B12 experience a significantly prolonged estrous cycle, reduced fertility compared to wild-type females, and a noticeable decrease in litter size. Additionally, abnormal formation of the mitotic spindle in immature follicles suggests a defect in cell meiotic division arrest in the ovaries of HSD17B12 heterozygotes [87]. Therefore, the transcription levels of the family genes would affect the mating behavior of animals. These family genes are linked to estradiol 17β-dehydrogenase, and these specific substances play a crucial role in the estrous behavior of female animals.Estradiol 17β-dehydrogenase is an enzyme that catalyzes the chemical reaction that converts estradiol-17β and NAD(P)^+^ to ketone and NAD(P)H^+^H^+^ [88]. During the estrous cycle of animals, estrogens (such as estradiol) play an important role, affecting sexual behavior and the development of reproductive organs [89, 90]. Therefore, the correlation between metabolites and mRNA in our urine may play a role in the mating process of animals, offering theoretical support that can be validated by future scholars.

In follicular fluid (Fig. 13), the mRNA associated with metabolites includes NOS3 and so on. The NOS3 gene encodes an enzyme called endothelial nitric oxide synthase (eNOS), which synthesizes nitric oxide (NO) in the organism. Nitric oxide is a biological signaling molecule involved in various physiological processes, including neural transmission, antimicrobial activity, anti-tumor activity, and reproductive processes [91]. Research suggests that different subtypes of NOS are found in the oviducts of mice and humans at various stages of the estrous and menstrual cycles by assessing NOS levels. Increased expression of iNOS has been observed in the oviducts of women with ectopic pregnancies [92]. The metabolite associated with NOS3 is Nitric-oxide synthase, a molecule that plays a particularly important role in the estrous cycle of female animals.

In summary, we have investigated the correlation between differential metabolites in urine, blood, and follicular fluid and the potentially targeted differential mRNA in the transcriptome of oocyte granulosa cells. This study provides a reference for future research on estrus-related studies in yaks.

## Conclusion

In conclusion, this study has identified differences in metabolites among three body fluids of female yaks after estrus treatment, shedding light on changes in body fluid metabolites during yak estrus and providing fundamental data for understanding the mechanisms of yak estrus in vivo. Furthermore, differentially expressed miRNAs and lncRNAs in follicular cells of female yaks have been identified, and a ceRNA regulatory network involving lncRNA-miRNA-mRNA has been constructed. Key genes associated with yak estrus have been discovered in this network, which may serve as potential biomarkers for future studies on yak estrus. These key genes are expected to provide valuable insights for further research in this field.

### Supplementary Information

**Supplementary Material 1.**

## Data Availability

Sequences are available from GenBank with the Bioproject accession number PRJNA958448 and PRJNA958176.
