# Correction: Transcriptomics and metabolomics of blood, urine and ovarian follicular fuid of yak at induced estrus stage

**DOI:** 10.1186/s12864-024-10223-3

**Published:** 2024-04-08

**Authors:** Huangqing Zhao, Yongzhen Huang, Shi Shu, Guowen Wang, Changqi Fu, Rong Huang, Jun Zhang, Huawei Su, Yang He, Chuzhao Lei, Lei Du, Jiahao Zhao, Wei Peng

**Affiliations:** 1 Qinghai University, Xining, China; 2 Northwest A&F University, Yangling, Shaanxi China; 3 China Agricultural University, Beijing, China

**Correction: BMC Genomics 25, 201 (2024)**

10.1186/s12864-024-10079-7

Following publication of the original article it was reported that the ‘Availability of data and materials’, ‘Funding’ and ‘Ethics approval and consent to participate’ declarations were missing. The correct declarations are given in this Correction and the original article has been updated.

**Funding** This research has been supported by the Key Technology Support Program of Qinghai Province (2021-ZJ-736).

**Data availability** Sequences are available from GenBank with the Bioproject accession number PRJNA958448 and PRJNA958176.

**Declarations** Ethics approval and consent to participate

The yaks in this study were all handled in strict accordance with the guidelines established by the Council for Animal Welfare of China. Additionally, the protocols for sample collection and animal handling were thoroughly reviewed and approved by the Faculty of Animal Policy and Welfare Committee of Northwest A&F University (FAPWCNWAFU, NWAFAC 1008). Furthermore, the study was carried out in compliance with the ARRIVE guidelines.

